# Analysis of Dento-Facial Parameters in the Young Population Using Digital Methods

**DOI:** 10.3390/diagnostics16030453

**Published:** 2026-02-01

**Authors:** Sonja Milosavljević, Milica Jovanović, Žaklina Rajković, Vladan Radisavljević, Tanja Šapić, Anđela Milojević Šamanović, Raša Mladenović, Vladan Đorđević, Milan Miljković, Danka Pajović, Jelena Todić, Marko Milosavljević

**Affiliations:** 1 Department of Dentistry, Faculty of Medical Sciences, University of Kragujevac, 34000 Kragujevac, Serbia; sonja.milosavljevic97@gmail.com (S.M.); zaklinakg19@gmail.com (Ž.R.); vradisavljevic097@gmail.com (V.R.); tanjasapic18@gmail.com (T.Š.); andjela-kg@hotmail.com (A.M.Š.); rasa.mladenovic@med.pr.ac.rs (R.M.); drvladandjordjevic@gmail.com (V.Đ.); drm.milosavljevic@yahoo.com (M.M.); 2 Research Centre for Biomedicine, Faculty of Medicine, University of Niš, 18000 Niš, Serbia; milandent89@yahoo.com; 3 Department of Prosthodontics, Institute of Dental Medicine, 34000 Kragujevac, Serbia; dankapajovic2115@gmail.com; 4 Department of Dentistry, Faculty of Medicine, University of Priština in Kosovska Mitrovica, 38220 Kosovska Mitrovica, Serbia; todic.j@gmail.com

**Keywords:** facial measurements, maxillary anterior teeth width, esthetics, dental photography

## Abstract

**Background/Objectives:** Facial and intraoral parameters are important guidelines in prosthetic planning and rehabilitation. This study aimed to analyze and determine the relationship between facial parameters and measurements on the upper anterior teeth using digital photography of the participants. **Methods:** This cross-sectional observational study included 82 student participants. Digital images (front facial and dental view) were taken of each participant, and then standardized images were used to measure facial and dental parameters. **Results:** The width of the maxillary anterior teeth and facial parameters were greater in males than in females, except for the medial canthus of the eye, which was slightly larger in females. A significant positive correlation was found between all facial parameters and the widths of the central and lateral incisors, as well as their combined sum. The strongest correlation was observed between the lateral canthus of the eye and the total width of the maxillary anterior teeth (r = 0.546; *p* < 0.001). In regression analysis, it was shown that the bizygomatic width had a statistically significant contribution to the prediction of the central incisor width (*p* = 0.045). It was also shown that the intraoral parameters, such as the height of the interdental papilla and interpapillary angle, are shape-dependent. Interincisal angles between the central incisors in all participants are significantly lower (*p* < 0.05) than the angles between incisal edges in other anterior teeth. **Conclusions:** Facial parameters cannot be used independently to predict dental parameters; nevertheless, when integrated with basic esthetic principles, they provide complementary information relevant to analytical procedures in restorative and prosthetic dentistry.

## 1. Introduction

Facial characteristics represent one of the most prominent determinants of perceived physical attractiveness, and numerous studies have emphasized the association between facial symmetry and esthetic perception [1,2]. Individuals considered physically attractive are often attributed positive personality traits, which may influence social interactions, partner selection, and professional opportunities. The long-standing debate on whether beauty is predominantly subjective or can be objectively quantified remains unresolved [3]. While certain authors underline the role of individual preferences and cultural background in shaping aesthetic judgments, others report relatively stable morphological indicators of facial attractiveness—such as symmetry, sexual dimorphism, and prototypical facial features—across different populations and methodological approaches in contemporary facial esthetics research [4].

One commonly applied approach to the quantification of facial harmony is based on mathematical proportions, most notably the Golden Ratio [5]. Recent studies suggest that the dimensions of maxillary anterior teeth, when evaluated in relation to selected facial measurements, may contribute to overall facial balance and esthetic perception, offering clinically useful reference values in restorative and prosthetic dentistry [6]. Nevertheless, findings regarding the influence of gender on dental proportions remain inconsistent, as some investigations report significant gender-related differences in dental parameters, whereas others fail to demonstrate such associations [7].

Anthropometric measurements are an integral component of diagnostic procedures and treatment planning for both extraoral and intraoral dental interventions. Despite their recognized importance, evidence indicates that standardized anthropometric data are not consistently applied in routine clinical practice. Yliallourodi et al. demonstrated that anthropometric values vary significantly according to gender and undergo changes with age [8]. More recent investigations have continued to examine the relevance of facial anthropometry in dental and craniofacial research, including large-scale studies identifying significant associations between facial dimensions and dental parameters such as occlusal vertical dimension, thereby supporting the continued relevance of anthropometric analysis in prosthodontic assessment [9,10]. In parallel, advances in three-dimensional facial imaging have revealed meaningful relationships between craniofacial soft tissue dimensions and demographic variables such as age and sex, highlighting ongoing methodological developments in this field [11].

Tooth height and width represent fundamental criteria in the selection of anterior teeth for prosthetic rehabilitation. While the clinical estimation of tooth width may be challenging, parameters such as arch form and smile line often facilitate the assessment of tooth height [12,13]. Various extraoral anthropometric measurements have been proposed as potential indicators of anterior tooth width; however, the reliability and consistency of individual parameters remain inconclusive [12]. In addition, contemporary research has explored innovative approaches, including machine learning models, to assess and predict facial dimensions based on dental parameters, further emphasizing the growing integration of facial and dental measurements in digital dentistry [14].

Considering that facial dimensions differ between males and females, a gender-specific perspective may be relevant when examining relationships between extraoral and intraoral anthropometric characteristics. Although studies involving broader age ranges are relatively limited [8,9,15,16,17,18], investigations conducted in young, homogeneous populations can provide valuable baseline data and methodological insights. Normative anthropometric data derived from young adult cohorts have previously been used to establish reference standards for facial and dental measurements, which may serve as a comparative framework for future research and clinical protocols [8,9].

Therefore, the aim of this study was to descriptively analyze facial and dental anthropometric parameters using two-dimensional digital methods and to explore their associations within a young student population. The objective was to provide population-specific, standardized anthropometric data and methodological observations that may serve as a reference for future research, rather than to establish predictive clinical models.

## 2. Materials and Methods

### 2.1. Study Design, Ethics Approval, and Sample Size Considerations

This cross-sectional observational study was conducted in accordance with the Declaration of Helsinki of 1975, which was revised in 2013. The study was conducted in the period between September 2024 and March 2025. Ethical approval was obtained from the Ethics Committee of the Faculty of Medical Sciences, University of Kragujevac (approval number: 01-14595/5; approval Date: 22 December 2023). Written informed consent was obtained from each participant.

The study sample consisted of dentistry students from the Faculty of Medical Sciences. A total of 82 participants were enrolled, including 62 females and 20 males, aged 21–35 years.

Sample size estimation was performed for planning purposes only. Assuming a small-to-moderate correlation (r ≈ 0.30), a two-tailed α level of 0.05, and 80% power, a minimum sample size of approximately 80 participants was estimated using G*Power 3.1.9.4 software. The final sample size (*n* = 82) is consistent with prior population-based anthropometric studies [19] and allows for preliminary assessment of associations while minimizing overinterpretation. This estimation was intended to support exploratory analyses rather than to test a predefined hypothesis or construct predictive models.

The study was conducted and reported in accordance with the STROBE (Strengthening the Reporting of Observational Studies in Epidemiology) guidelines [20] (Table S1).

### 2.2. Inclusion and Exclusion Criteria

Inclusion criteria of the study were participants over 18 years with Angle Class I occlusion. Eligible participants had healthy teeth without anomalies in number, shape, or size, and without mobility, crowding, or periodontal disease.

Exclusion criteria of the study included loss of tooth substance due to caries, abrasion, or erosion. Also excluded were participants with crowns or bridges in the intercanine region; absence of anterior maxillary teeth. Other exclusions were those undergoing orthodontic treatment during and before the study, or participants with pronounced facial asymmetry, congenital anomalies, or facial trauma.

### 2.3. Data Collection and Image Processing

**Figure 1:**
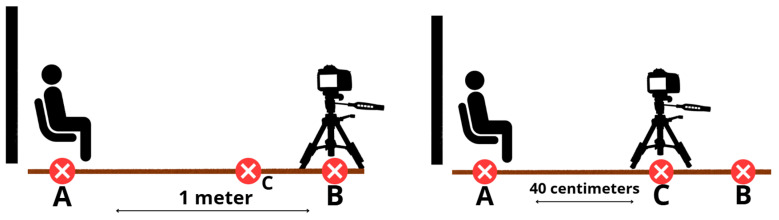
Standardized photographic setup of participant and camera.

All frontal photographs were taken by a single examiner (S.M.) using a Canon EOS 800D digital camera (Canon Inc., Ōta, Tokio, Japan) with a 100 mm macro lens mounted on a tripod. To standardize the photographing procedure, three positions were marked on the floor:

- Position A: the participant’s position,
- Position B: the photographer’s position at 1 m from the participant (for facial photographs),
- Position C: the photographer’s position at 40 cm from the participant (for dental photographs) (Figure 1).

All photographs were obtained under standardized conditions. Facial photographs were taken under natural daylight against a neutral white background to ensure uniform illumination and minimize shadows and color distortion. No additional artificial lighting (e.g., reflectors or lighting panels) was used. All participants were photographed at a fixed distance using identical camera settings.

Head position and stability were standardized during image acquisition. Horizontal and vertical rulers were positioned adjacent to the participant’s head to enable image calibration and orientation control. In addition, a laser beam parallel to the floor was used to ensure correct head positioning. The projection of the laser beam onto the participant’s head was used to align the Frankfurt horizontal plane—defined as the line connecting the lowest point of the orbital rim and the highest point of the external auditory canal—parallel to the horizontal plane. Correct alignment was visually verified by two independent operators before image capture.

Participants were instructed to maintain an upright posture, a neutral facial expression, and to look directly at the camera. Photographs were taken only after confirmation of correct head orientation and stability. Images were acquired both in a relaxed facial posture and while smiling. When zenith points were not clearly visible during smiling, dental retractors were used to improve visualization.

After establishing optimal photographic conditions, soft-tissue landmarks were physically marked on the participants’ faces before image acquisition to ensure accurate and consistent landmark identification during subsequent digital analysis.

For each recording position, multiple photographs were taken, and the highest-quality images were selected for further digital processing. All facial photographs were captured at a resolution of 300 dpi to ensure sufficient detail for standard facial measurements, while dental photographs were obtained at higher resolutions (up to 600 dpi) to allow precise visualization of fine anatomical structures. Image calibration and scaling were performed using CorelDRAW Graphics Suite 22.0 by converting pixel distances into millimeters based on horizontal and vertical reference rulers positioned adjacent to the participant’s head. Following calibration, additional image processing was performed in GIMP (version 2.10.38), where a reference grid was applied to facilitate precise measurement of tooth width and height in the middle third of the crown. After file export, all parameters were analyzed and digitally measured using IC Measure software 4.0.0.219, ensuring accurate, reproducible, and standardized measurements.

**Figure 2:**
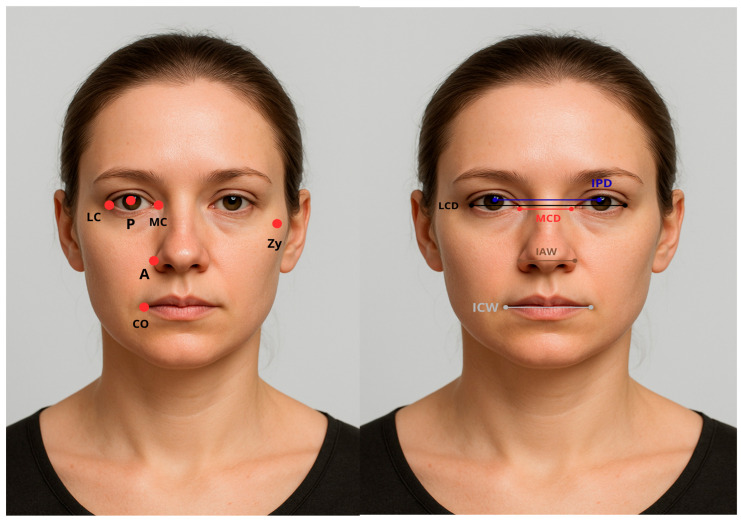
Soft tissue points and Facial landmarks.

Points of soft tissue and facial landmarks that are used and measured in the study are explained in Table 1 and Table 2, and are colorized and presented in Figure 2. A generative AI tool (OpenAI ChatGPT, version 4.0) was used exclusively to generate an artificial human face for Figure 2. The purpose of using an AI-generated image was to ensure full protection of participant identity, because several anatomical reference points used in the cephalometric analysis are located in the periocular region. No images of actual participants were used. The AI-generated face served only for illustrative purposes to demonstrate landmark positions, without influencing data collection, measurements, or results.

**Figure 3:**
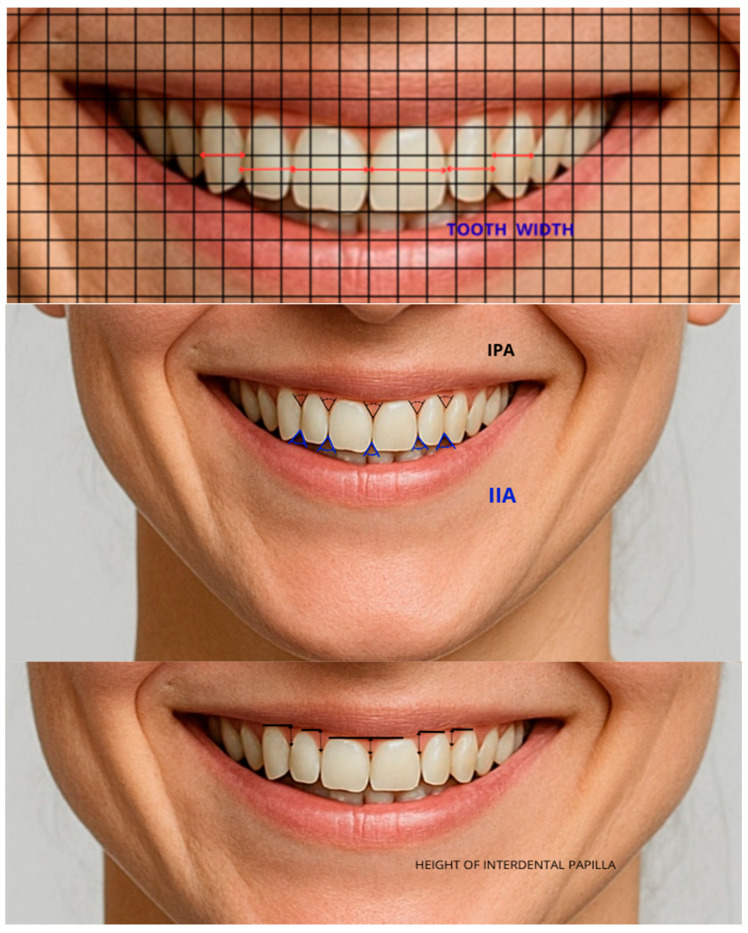
Dental landmarks.

Table 3 and Figure 3 explain and present the dental landmarks that are measured in the study.

**Figure 4:**
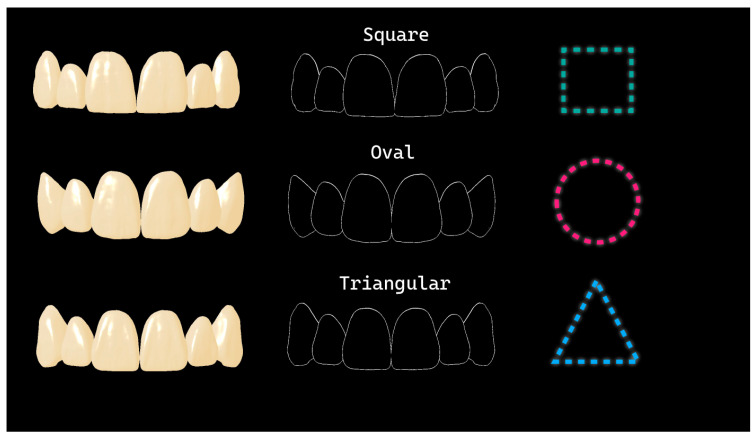
Shapes of the upper anterior teeth.

Based on Williams’ tooth shape classification, the teeth were categorized into three basic forms [21]. A tooth was classified as square if its mesial and distal surfaces were parallel to one another; triangular if the surfaces converged slightly toward the cervical region; and oval if they converged toward both the cervical and incisal regions (Figure 4) [22].

### 2.4. Measurement Reliability and Validity

The measurements were performed by a single examiner (S.M.). To assess intra-examiner reliability, 30 subjects were randomly selected from the total sample. The same examiner repeated all measurements on the same photographs after several days, using an identical procedure for landmark identification and measurement. The Intraclass Correlation Coefficient (ICC) was used to evaluate the agreement between the first and second measurements. Two variables were selected as representative of all measurements—one linear variable (distance between reference points) and one angular variable (angle between reference lines). For the linear variable, the ICC was 0.923 (95% CI: 0.845–0.963), while for the angular variable, the ICC was 0.931 (95% CI: 0.860–0.966), indicating excellent intra-examiner reliability.

### 2.5. Statistical Analysis

Statistical analyses were performed for exploratory purposes to describe associations rather than to establish predictive relationships. The collected data were analyzed using SPSS software version 24.0 (SPSS Inc., Chicago, IL, USA). Statistical significance was set at *p* < 0.05. Descriptive statistics were performed for both categorical and continuous variables, and results are presented as mean ± standard deviation (SD). Parametric tests were retained for robustness, given the group sizes (male = 20; female = 62). The Shapiro–Wilk test was applied to assess normality, and Welch’s *t*-test was used when variances were unequal. Despite the moderately small number of male participants, the distributions did not exhibit extreme skewness or outliers that would necessitate the use of non-parametric tests. Independent *t*-tests, one-way ANOVA, and regression analyses were used to compare mean values across dependent and independent variables. Tamhane’s post hoc test was applied when the assumption of homogeneity of variances was violated. Pearson’s correlation coefficient (r) was used to evaluate the relationship between facial parameters and the widths of the maxillary anterior teeth.

## 3. Results

A total of 82 participants, aged from 21 to 35 years old, with a mean age and SD of the study sample of 24.88 ± 2.891, participated in the study. Of the 82 participants, 62 (75.6%) were female, and 20 (24.4%) were male.

### 3.1. Teeth Parameters

Table 4 shows the width of the upper anterior teeth (for each tooth separately), as well as the sum of the widths of the central incisors (CIW), central and lateral incisors (CLIW), and all anterior teeth, expressed as the intercanine distance (ICD).

There was no significant difference in mesiodistal tooth width between genders, except for the width of the right canines, which was higher in male participants. 

### 3.2. Facial Parameters

Among extraoral measured variables, there were statistically significant differences between genders in BZD and IAW, while other parameters were similar in males and females (Table 5).

Correlation analyses between facial measurements and tooth widths (Table 6) revealed generally weak to moderate positive associations. In particular, BZD demonstrated a moderate correlation with the widths of central incisors, whereas IAW showed weaker correlations. Similar patterns were observed for lateral incisors. Negative correlations were noted between all extraoral parameters and canine widths. Given the large number of associations examined, these analyses are considered exploratory, and caution is warranted when interpreting statistical significance due to potential type I error.

The multivariate regression analysis of the width of anterior teeth (CIW, CLIW, ICD) and independent variables (BZD, IAW, ICW, IPD, LCD, MCD, gender) is shown in Table 7. Variance inflation factors (VIFs) were checked for all independent variables in the regression models, and were all below five, indicating no problematic multicollinearity.

For CIW, the model explained 24% of the variance (Adjusted R^2^ = 0.241, F = 4.669, *p* < 0.001). Among predictors, only BZD reached statistical significance (β = 0.398, *p* = 0.045), suggesting a positive association within this exploratory framework.

For CLIW, the model explained 26.1% of the variance (Adjusted R^2^ = 0.261, F = 5.086, *p* < 0.001). No single predictor was statistically significant, although BZD had the largest beta coefficient (0.296), indicating the most consistent, but non-significant, association.

For IPD, the model explained 14.1% of the variance (Adjusted R^2^ = 0.141, F = 2.903, *p* = 0.01). All predictors were non-significant, with the largest beta coefficients observed for LCD (0.224) and BZD (0.197). The relatively low adjusted R^2^ values indicate limited explanatory power and do not support predictive clinical application.

These regression analyses were performed for exploratory purposes only and are not intended to establish predictive models.

### 3.3. Soft Tissue Parameters

The values of HIP and IPA are presented in Table 8. Both HIP and IPA measured between the central incisors were statistically significantly higher in male participants.

By comparing the values of IPA and HIP between the whole anterior teeth in all subjects, a one-factor ANOVA with repeated measurements was conducted. To control the type I error in multiple pairwise comparisons, the Holm–Bonferroni correction was used. Formal testing of sphericity was not performed, which represents a limitation of this analysis; however, the distributions did not show extreme skewness or outliers, and the corrections applied provide a robust evaluation of differences. Significant difference between values of IPA is determined among the next angles: IPA 1 vs. IPA 3 (*p* ˂ 0.001), IPA 2 vs. IPA 5 (*p* ˂ 0.001), IPA 1 vs. IPA 4 (*p* ˂ 0.001), IPA 1 vs. IPA 5 (*p* ˂ 0.001), and IPA 2 vs. IPA 3 (*p* = 0.003). On the other hand, significant difference between HIP values is determined among next variables: HIP 1 vs. HIP 2 (*p* ˂ 0.001), HIP 1 vs. HIP 4 (*p* ˂ 0.001), HIP 2 vs. HIP 3 (*p* ˂ 0.001), HIP 2 vs. HIP 4 (*p* ˂ 0.001), and HIP 2 vs. HIP 5 (*p* ˂ 0.001). These findings show that zenith points on central incisors are at a similar distance to zeniths of canines, while zeniths of lateral incisors are lower.

Additionally, tooth shape influenced these soft-tissue parameters: square central incisors exhibited higher IPA, while triangular central incisors showed higher HIP (Table 9).

These findings describe morphological patterns in this cohort and should be interpreted within the context of exploratory, population-specific observations rather than as prescriptive clinical norms.

### 3.4. Interincisal Angles

In addition to the previously analyzed parameters, this study also examined the angles formed between the incisal edges of the anterior teeth, which are important for assessing the esthetic characteristics of a smile. The values of these angles are presented in Table 10. No statistically significant differences were found between genders, although females showed slightly higher values. In contrast, the angle between the central incisors in the total sample was significantly smaller (*p* < 0.05) than the angles measured between other anterior teeth. Using one-way repeated-measures ANOVA with Holm–Bonferroni correction, the following adjusted *p*-values were obtained: IIA1 vs. IIA2: *p* < 0.001; IIA1 vs. IIA3: *p* < 0.001; IIA1 vs. IIA4: *p* = 0.004; IIA1 vs. IIA5: *p* < 0.001. These measurements provide descriptive data on anterior incisal geometry in a young adult population and represent some of the first reported photographic values for these angles.

When comparing the IIA between central incisors across different tooth shapes, no statistically significant differences were observed. This indicates that tooth shape does not influence the magnitude of the incisal–incisal angle.

## 4. Discussion

In the present exploratory cross-sectional study, facial and dental anthropometric parameters were analyzed using standardized two-dimensional digital photography in a young adult student population. The observed gender-related differences, including greater right canine width, BZD, IAW, and selected soft-tissue parameters (IPA and HIP between the central incisors) in male participants, are generally consistent with previously reported patterns of sexual dimorphism in craniofacial and dental morphology [19,23,24,25,26,27,28]. However, given the relatively small and gender-imbalanced sample, particularly the limited number of male participants, these findings should be interpreted cautiously and primarily as descriptive observations rather than definitive population characteristics.

Correlation analyses revealed weak to moderate associations between selected extraoral facial parameters and the widths of central, lateral, and total anterior maxillary teeth. These results suggest that facial anthropometric measurements may provide contextual information when evaluating anterior tooth dimensions, but their limited strength indicates that they cannot serve as reliable standalone indicators. The present findings reinforce the notion that extraoral measurements should be interpreted as supportive reference points within a multifactorial diagnostic framework rather than as direct determinants of dental dimensions.

The multiple regression analyses performed in this study were exploratory in nature and aimed to assess potential associations rather than to establish predictive clinical models. Among the evaluated variables, bizygomatic width (BZD) emerged as the only parameter showing a statistically significant association with central incisor width, while other facial parameters did not demonstrate independent predictive value. The modest proportion of explained variance further underscores the limited explanatory capacity of individual facial measurements. These results are consistent with previous studies reporting population-specific variability and methodological heterogeneity in craniofacial–dental relationships [23,29,30,31,32]. For example, Mishra et al. identified several predictors of maxillary anterior tooth width, including BZD, IAW, ICW, and age [23], while Attokaran et al. reported stronger correlations between IAW and total anterior tooth width but emphasized that such parameters should not be used in isolation [30].

The moderate association observed between BZD and anterior tooth width in this cohort may partially reflect craniofacial growth patterns and soft-tissue maturation processes that continue into early adulthood and generally stabilize between 18 and 25 years of age [33]. Nevertheless, given the narrow age range of the participants, the present data primarily reflect a specific developmental stage and should not be extrapolated to older or clinically heterogeneous populations.

Embryogenetic concepts, such as Gerber’s theory linking nasal morphology and maxillary incisor position through their common origin in the frontonasal process, provide a theoretical basis for examining relationships between facial and dental dimensions [34]. Previous studies have demonstrated associations between parameters such as IAW and ICW and the width of maxillary anterior teeth [35]. While the present findings show correlations in a similar direction, their moderate magnitude and limited predictive value highlight the influence of population-specific characteristics and methodological factors. Consequently, facial anthropometric parameters should be integrated with additional objective and subjective criteria during prosthetic planning rather than applied as universal rules.

Interpupillary distance (IPD) was greater in male participants, in agreement with anthropometric data reported for European and Middle Eastern populations [31,36,37]. Although IPD is considered relatively stable in adulthood and has been proposed as a reference parameter in esthetic dentistry [38], the variability observed across individuals limits its clinical applicability when used alone. As with other extraoral measurements, IPD should be regarded as an adjunctive reference rather than a determinant of anterior tooth selection.

Collectively, these findings emphasize the importance of population-specific anthropometric datasets and confirm that standardized measurements, while informative, cannot replace individualized clinical assessment—particularly when estimating the total width of maxillary anterior teeth in the absence of pre-extraction records.

Facial analysis remains a fundamental component of both fixed and removable prosthetic planning. With the increasing integration of digital technologies in dentistry, standardized facial photography has become a practical and accessible tool for anthropometric assessment [39,40]. When combined with basic esthetic principles, angular and linear facial measurements can contribute to treatment planning; however, the absence of a universally reliable method for artificial tooth selection remains well documented [41,42].

The distribution pattern of mesiodistal anterior tooth widths observed in this study—widest central incisors, followed by canines and lateral incisors—is consistent with established dental norms [43]. Although males exhibited larger mean dimensions, the degree of sexual dimorphism varied compared to previous studies conducted in European and Asian populations [19,23,25,26,27,28]. This variability underscores the influence of genetic, developmental, and ethnic factors and supports the need for regional reference data in prosthetic dentistry.

Sexual dimorphism in maxillary canines, observed in this and previous investigations [26,27,28], may be related to prolonged enamel formation during amelogenesis in males. While this biological explanation provides context for dimensional differences, its direct translation into clinical decision-making should be approached with caution, particularly in diverse patient populations.

Regarding soft-tissue parameters, HIP and IPA values differed between genders and varied according to tooth position and shape. Higher papilla height between triangular-shaped incisors and wider interproximal angles associated with square-shaped teeth are consistent with earlier reports [44,45]. However, discrepancies across studies suggest that these parameters are influenced by multiple interacting factors, including tooth morphology, gingival biotype, and individual variation. Accordingly, the present findings should be interpreted as descriptive patterns within this cohort rather than normative standards.

Analysis of interincisal angles revealed a gradual increase from central incisors to canines, with no significant gender-related differences. Although these angular relationships have been described conceptually in the literature [46], quantitative photographic data remain limited. The present results contribute descriptive values for a young adult population, while recognizing that sample size and gender imbalance may have influenced the observed distributions.

Beyond objective measurements, smile esthetics are strongly influenced by subjective perception, which is shaped by cultural, ethnic, and individual factors [47]. Variables such as gender, age, education level, and socioeconomic background have been shown to affect esthetic evaluation, with females and individuals with higher educational attainment often demonstrating greater sensitivity to smile characteristics [48]. The influence of age on smile perception remains debated, with some studies suggesting increased evaluative complexity in older individuals, while others report minimal effects [49,50]. These findings reinforce the concept that anthropometric data should complement, rather than replace, subjective patient-centered assessment.

Study limitations: This study is limited by its relatively small, homogeneous sample consisting exclusively of young dental students with Angle Class I occlusion, which does not represent the broader prosthodontic patient population. The unequal gender distribution, single-center design, and reliance on two-dimensional photographic analysis for inherently three-dimensional structures further restrict generalizability. Consequently, the results should be interpreted as exploratory and population-specific. Future studies incorporating larger, age-diverse samples and three-dimensional digital imaging techniques are necessary to further clarify facial–dental relationships and their potential clinical relevance.

## 5. Conclusions

The present study provides a descriptive analysis of facial and dental anthropometric parameters obtained using two-dimensional digital photographic methods in a young adult student population. The findings demonstrate the presence of weak to moderate associations between selected extraoral facial measurements and the dimensions of maxillary anterior teeth; however, the observed relationships showed limited explanatory power and should be interpreted with caution.

The regression analyses performed in this study were exploratory in nature and did not identify robust or clinically predictive models for anterior tooth dimensions. Although certain facial parameters, particularly bizygomatic width, showed more consistent associations with anterior tooth widths, these findings do not support the use of extraoral anthropometric measurements as standalone predictors in prosthetic or restorative treatment planning.

The analysis of soft tissue parameters and interincisal angles revealed consistent intra-arch patterns, including variations related to tooth position and shape. These observations may contribute to a better descriptive understanding of smile characteristics in young adults, but should not be extrapolated beyond the investigated population.

Rather than proposing clinical decision rules, the findings contribute population-specific descriptive data and highlight methodological considerations relevant to digital anthropometric analysis. These results may serve as a reference framework for future research employing larger, age-diverse samples and three-dimensional imaging techniques.

The study underscores that anthropometric measurements should be interpreted within a broader morphological and subjective context and should not replace individualized clinical assessment.

## Figures and Tables

**Table 1:** Explanation of soft tissue points.

Soft Tissue Points	Description
Pupil (P)	The center of the pupil
Lateral eye canthus (LC)	The outer point of the eye
Medial eye canthus (MC)	The inner point of the eye
Zygion (Zy)	The most lateral soft tissue points of the zygomatic arch
Alar (A)	The outer point of the alar
Lip commissure (CO)	The outer point of the lip

**Table 2:** Explanation of facial landmarks measured in the study.

Facial Landmarks	Description
Lateral canthus of the eye distance (LCD)	Right LC–Left LC
Medial canthus of the eye distance (MCD)	Right MC–Left MC
Interpupillary distance (IPD)	Right P–Left P
Interalar width (IAW)	Right A–Left A
Intercommissural width (ICW)	Right CO–Left CO
Bizygomatic distance (BZD)	Right Zy–Left Zy

**Table 3:** Explanation of dental landmarks measured in the study.

Dental Landmarks	Description
Tooth Width	Distance between the mesial and distal contact points of a given tooth
Interpapillary Angle (IPA)	The angle formed by the papilla of two adjacent teeth, with its apex facing incisally and open towards gingivally
Interincisal Angle (IIA)	The angle formed by the incisal edges of two adjacent teeth, with its apex facing gingivally and open towards incisally
Height of interdental papilla (HIP)	Distance from the tip of the papilla to the tangent passing through the zenith of the given tooth

**Table 4:** Comparison of the mean (SD) of age (years) and width (mm) of upper anterior teeth between genders.

Characteristics	Participants (*n* = 82)	Male (*n* = 20)	Female (*n* = 62)	*p* Value (Between Gender)	Mean Difference	95%CI	
						Lower	Upper
Age	24.88 ± 2.891	25.05 ± 3.120	24.50 ± 2.733	0.056	1.550	−0.044	3.144
Right central incisor width	8.980 ± 0.694	9.005 ± 0.666	8.973 ± 0.708	0.853	0.0324	−0.3216	0.3864
Right lateral incisor width	6.137 ± 0.672	6.220 ± 0.583	6.110 ± 0.701	0.489	0.1103	−0.2095	0.4301
Right canine width	6.763 ± 0.578	6.910 ± 0.487	6.569 ± 0.454	0.010 *	0.3406	0.0889	0.5924
Left central incisor width	9.015 ± 0.699	9.115 ± 0.584	8.982 ± 0.734	0.413	0.1327	−0.1916	0.4571
Left lateral incisor width	6.067 ± 0.665	6.165 ± 0.612	6.035 ± 0.683	0.430	0.1295	−0.2121	0.4711
Left canine width	6.851 ± 0.554	6.925 ± 0.498	6.681 ± 0.454	0.061	0.2444	0.0064	0.4823
CIW	17.995 ± 1.355	18.120 ± 1.189	17.955 ± 1.411	0.610	0.1652	−0.4842	0.8145
CLIW	30.198 ± 2.230	30.505 ± 1.857	30.1 ± 2.341	0.429	0.4082	−0.6239	1.4403
ICD	43.591 ± 2.345	44.34 ± 2.046	43.35 ± 2.398	0.080	0.990	−0.1235	2.1035

Variables are presented as mean, and standard deviation; 95%CI—Confidence Interval; * *p* value ˂ 0.05 was considered significant; Welch’s *t*-test.

**Table 5:** Comparison of the mean (SD) of extraoral measured parameters (mm) between genders.

Facial Variable	Participants (*n* = 82)	Male (*n* = 20)	Female (*n* = 62)	*p* Value (Between Gender)	Mean Difference	95%CI	
						Lower	Upper
BZD	142.91 ± 24.067	152.29 ± 6.579	144.547 ± 7.511	<0.001 *	7.9545	4.4024	11.5067
IAW	36.54 ± 3.23	39.22 ± 3.502	35.676 ± 2.631	<0.001 *	3.5442	1.7949	5.2935
ICW	51.822 ± 3.95	53.755 ± 5.208	51.198 ± 3.264	0.05	2.5566	0.0051	5.1081
LCD	89.763 ± 5.189	89.880 ± 6.043	89.726 ± 4.937	0.918	0.1542	−2.8990	3.2074
MCD	32.106 ± 3.232	31.985 ± 3.141	32.145 ± 3.285	0.846	−0.1602	−1.8217	1.5014
IPD	64.074 ± 5.643	65.225 ± 4.418	63.703 ± 5.969	0.228	1.5218	−0.9891	4.0327

Variables are presented as mean, and standard deviation; 95%CI—Confidence Interval; * *p* value ˂ 0.05 was considered significant; Welch’s *t*-test.

**Table 6:** Correlation analysis between extraoral parameters and the width of upper anterior teeth.

Variable (Whole Participants *n* = 82)	Right Central Incisor Width	Left Central Incisor Width	Right Lateral Incisor Width	Left Lateral Incisor Width	Right Canine Width	Left Canine Width	CIW	CLIW	ICD
BZD	r = 0.432 *p* < 0.001 *	r = 0.464 *p* < 0.001 *	r = 0.390 *p* < 0.001 *	r = 0.295 *p* < 0.001 *	r = −0.043 *p* = 0.699	r = −0.149 *p* = 0.180	r = 0.461 *p* < 0.001 *	r = 0.486 *p* < 0.001 *	r = 0.423 *p* < 0.001 *
	95% Confidence interval (lower-upper)								
	0.237–0.593	0.275–0.619	0.189–0.560	0.083–0.481	−0.285–0.176	−0.355–0.07	0.271–0.616	0.301–0.636	0.227–0.586
IAW	r = 0.321 *p* = 0.003 *	r = 0.323 *p* = 0.003 *	r = 0.354 *p* = 0.001 *	r = 0.338 *p* = 0.002 *	r = −0.036 *p* = 0.749	r = −0.137 *p* = 0.220	r = 0.331 *p* = 0.002 *	r = 0.409 *p* < 0.001 *	r = 0.354 *p* = 0.001 *
	95% Confidence interval (lower-upper)								
	0.112–0.503	0.114–0.505	0.148–0.530	0.131–0.517	−0.251–0.182	−0.344–0.082	0.123–0.511	0.211–0.575	0.148–0.53
ICW	r = 0.421 *p* < 0.001 *	r = 0.412 *p* < 0.001 *	r = 0.385 *p* < 0.001 *	r = 0.328 *p* = 0.003 *	r = −0.142 *p* = 0.204	r = −0.236 *p* = 0.033 *	r = 0.428 *p* < 0.001 *	r = 0.474 *p* < 0.001 *	r = 0.374 *p* = 0.001 *
	95% Confidence interval (lower-upper)								
	0.224–0.585	0.214–0.577	0.183–0.556	0.119–0.509	−0.348–0.077	−0.431–−0.02	0.233–0.590	0.286–0.627	0.171–0.547
LCD	r = 0.423 *p* < 0.001	r = 0.464 *p* < 0.001	r = 0.410 *p* < 0.001	r = 0.299 *p* = 0.006	r = −0.159 *p* = 0.153	r = −0.190 *p* = 0.087	r = 0.457 *p* < 0.001 *	r = 0.490 *p* < 0.001 *	r = 0.546 *p* < 0.001 *
	95% Confidence interval (lower-upper)								
	0.227–0.586	0.275–0.619	0.212–0.576	0.088–0.485	−0.363–0.06	−0.391–0.028	0.266–0.613	0.305–0.639	0.373–0.682
MCD	r = 0.218 *p* = 0.049	r = 0.263 *p* = 0.017	r = 0.366 *p* = 0.001	r = 0.249 *p* = 0.024	r = −0.123 *p* = 0.271	r = −0.148 *p* = 0.184	r = 0.247 *p* = 0.025 *	r = 0.335 *p* = 0.002 *	r = 0.397 *p* < 0.001 *
	95% Confidence interval (lower-upper)								
	0.001–0.415	0.049–0.454	0.162–0.540	0.034–0.442	−0.331–0.097	−0.354–0.071	0.032–0.440	0.127–0.515	0.197–0.565
IPD	r = 0.265 *p* = 0.016 *	r = 0.292 *p* = 0.008 *	r = 0.290 *p* = 0.008 *	r = 0.145 *p* = 0.193	r = −0.119 *p* = 0.288	r = −0.124 *p* = 0.266	r = 0.287 *p* = 0.009 *	r = 0.305 *p* = 0.005 *	r = 0.265 *p* = 0.016 *
	95% Confidence interval (lower-upper)								
	0.051–0.456	0.08–0.479	0.078–0.477	−0.074–0.351	−0.328–0.101	−0.332–0.096	0.075–0.474	0.094–0.490	0.051–0.456

Pearson’s coefficient of correlation (r); * *p* value ˂ 0.05 was considered significant.

**Table 7:** The multivariate regression analysis for BZD, IAW, ICW, IPD, LCD, MCD, and gender with the sum of the width of the anterior teeth (CIW, CLIW, and ICD).

		Unstandardized Coefficients		Standardized Coefficients	t	*p* Value	95.0% Confidence Interval for B	
		B	Std. Error	Beta			Lower Bound	Upper Bound
CIW	(Constant)	0.712	3.348		0.213	0.832	−5.960	7.383
	BZD	0.067	0.033	0.398	2.038	0.045 *	0.002	0.133
	IAW	0.034	0.076	0.080	0.442	0.660	−0.118	0.186
	ICW	0.026	0.059	0.077	0.448	0.656	−0.091	0.143
	LCD	0.053	0.055	0.201	0.948	0.346	−0.058	0.163
	MCD	−0.112	0.064	−0.267	−1.736	0.087	−0.240	0.017
	IPD	0.040	0.035	0.168	1.163	0.249	−0.029	0.110
	Gender	0.644	0.432	0.205	1.492	0.140	−0.216	1.505
CLIW	(Constant)	2.449	5.432		0.451	0.653	−8.375	13.273
	BZD	0.082	0.054	0.296	1.540	0.128	−0.024	0.189
	IAW	0.116	0.124	0.168	0.938	0.351	−0.131	0.363
	ICW	0.054	0.095	0.096	0.569	0.571	−0.136	0.244
	LCD	0.069	0.090	0.161	0.767	0.445	−0.110	0.248
	MCD	−0.093	0.105	−0.134	−0.885	0.379	−0.301	0.116
	IPD	0.059	0.056	0.150	1.054	0.295	−0.053	0.172
	Gender	0.914	0.701	0.177	1.304	0.196	−0.483	2.310
ICD	(Constant)	23.152	6.161		3.758	0.000	10.876	35.428
	BZD	0.058	0.061	0.197	0.948	0.346	−0.063	0.179
	IAW	0.068	0.140	0.094	0.487	0.628	−0.212	0.348
	ICW	0.023	0.108	0.039	0.213	0.832	−0.192	0.238
	LCD	0.101	0.102	0.224	0.991	0.325	−0.102	0.305
	MCD	−0.071	0.119	−0.098	−0.601	0.550	−0.308	0.165
	IPD	0.028	0.064	0.068	0.444	0.658	−0.099	0.156
	Gender	−0.161	0.795	−0.030	−0.202	0.840	−1.745	1.423

Beta—denotes the correlation between dependent and independent variables; B—unstandardized coefficient, i.e., average estimation of BZD, IAW, ICW, LCD, MCD, IPD, and gender with CIW, CLIW, ICD; *t*-test of the regression coefficient; * *p* value ˂ 0.05 was considered significant.

**Table 8:** Comparison of mean (SD) interdental papilla height (mm) and interpapillary angles (degrees) between genders in the upper anterior teeth.

Characteristics	Participants (*n* = 82)	Male (*n* = 20)	Female (*n* = 62)	*p* Value (Between Gender)	Mean Difference	95%CI	
						Lower	Upper
HIP 1 (between central incisors)	6.950 ± 1.903	7.770 ± 1.579	6.685 ± 1.934	0.016 *	1.0845	0.2143	1.9547
HIP 2 (between right central and lateral incisor)	5.927 ± 1.475	6.420 ± 1.347	5.768 ± 1.489	0.075	0.6523	−0.0696	1.3742
HIP 3 (between right lateral incisor and canine)	6.635 ± 1.804	7.095 ± 2.062	6.487 ± 1.705	0.243	0.6079	−0.4361	1.6519
HIP 4 (between left central and lateral incisor)	6.240 ± 1.357	6.585 ± 1.512	6.129 ± 1.297	0.235	0.4560	−0.3139	1.2259
HIP 5 (between left lateral incisor and canine)	6.657 ± 1.754	6.980 ± 2.044	6.553 ± 1.655	0.404	0.4268	−0.6047	1.4582
IPA 1 (between central incisors)	62.144 ± 16.136	56.950 ± 11.424	63.819 ± 17.128	0.046 *	−6.8694	−13.6134	−0.1253
IPA 2 (between right central and lateral incisor)	56.888 ± 12.250	55.040 ± 11.557	57.484 ± 12.498	0.426	−2.4439	−8.7289	3.8412
IPA 3 (between right lateral incisor and canine)	52.324 ± 11.194	50.690 ± 10.400	52.852 ± 11.467	0.436	−2.1616	−7.7314	3.4081
IPA 4 (between left central and lateral incisor)	54.633 ± 10.936	51.035 ± 10.274	55.794 ± 10.969	0.085	−4.7585	−10.2184	0.7013
IPA 5 (between left lateral incisor and canine)	51.344 ± 9.696	52.710 ± 8.850	50.903 ± 9.982	0.447	1.8068	−2.9603	6.5738

Variables are presented as mean, and standard deviation; 95% CI—Confidence Interval; * *p* value ˂ 0.05 was considered significant; Welch’s *t*-test.

**Table 9:** Comparison of mean (SD) interpapillary angles (degrees) and interdental papilla height (mm) between central incisors with different tooth shapes.

Variable	Oval Shape (*n* = 18)	Triangular Shape (*n* = 23)	Square Shape (*n* = 41)	*p* Value
IPA between central incisors	65.878 ± 20.345	52.357 ± 11.439	65.995 ± 14.240	Oval vs. triangular *p* = 0.054
				Oval vs. square *p* = 1.000
				Triangular vs. square *p* ˂ 0.001 *
HIP between central incisors	6.594 ± 1.962	6.337 ± 1.502	8.322 ± 1.866	Oval vs. triangular *p* = 0.021 *
				Oval vs. square *p* = 0.947
				Triangular vs. square *p* ˂ 0.001 *

Variables are presented as mean and standard deviation; * *p* value ˂ 0.05 was considered significant; One-way ANOVA; Post hoc Tamhane test.

**Table 10:** Comparison of mean (SD) angles between incisal edges of the upper anterior teeth (degrees) between genders.

Variable (Angle Between Incisal Edges)	Participants (*n* = 82)	Male (*n* = 20)	Female (*n* = 62)	*p* Value (Between Gender)	Mean Difference	95%CI	
						Lower	Upper
IIA 1 (Central incisors)	50.341 ± 21.137	52.430 ± 24.543	49.668 ± 20.094	0.652	2.7623	−9.6414	15.1659
IIA 2 (Right central and lateral incisor)	61.157 ± 21.863	58.245 ± 19.103	62.097 ± 22.746	0.460	−3.8518	−14.2922	6.5887
IIA 3 (Right lateral incisor and canine)	75.911 ± 19.189	75.315 ± 20.230	76.103 ± 19.009	0.879	−0.7882	−11.2515	9.6750
IIA 4 (Left central and lateral incisor)	58.512 ± 18.258	57.365 ± 18.259	58.882 ± 18.391	0.749	−1.5173	−11.0939	8.0594
IIA 5 (Left lateral incisor and canine)	68.743 ± 19.306	70.930 ± 20.031	68.037 ± 19.179	0.575	2.8929	−7.5051	13.2909

Variables are presented as mean, and standard deviation; 95%CI—Confidence Interval.

## Data Availability

The original contributions presented in this study are included in the article. Further inquiries can be directed to the corresponding author.

## Supplementary Materials

The following supporting information can be downloaded at: https://www.mdpi.com/article/10.3390/diagnostics16030453/s1, Table S1: STROBE Statement—Checklist.

## Abbreviations

The following abbreviations are used in this manuscript:

P	Pupil
LC	Lateral eye canthus
MC	Medial eye canthus
Zy	Zygion
LCD	Lateral canthus of the eye distance
MCD	Medial canthus of the eye distance
IPD	Interpupillary distance
IAW	Interalar width
ICW	Intercommissural width
BZD	Bizygomatic distance
CIW	Width of central incisors
CLIW	Width of central and lateral incisors
ICD	Intercanine distance
IPA	Interpapillary Angle
IIA	Interincisal Angle
HIP	Height of interdental papilla
