# Supplementary material for: Analysis of Dento-Facial Parameters in the Young Population Using Digital Methods
Source: Diagnostics (Basel). 2026 Feb 1;16(3):453. doi: 10.3390/diagnostics16030453 (PMC12897327; doi:10.3390/diagnostics16030453)
Supplement: Supplementary file 1 [file diagnostics-16-00453-s001.zip › diagnostics-4048828-supplementary.pdf]

STROBE Statement—Checklist of items that should be included in reports of ***cross-sectional studies***

	**Item No**	**Recommendation**	**Section/Page**
**Title and abstract**	1	(*a*) Indicate the study's design with a commonly used term in the title or the abstract	Title and Abstract (Title includes „cross-sectional observational study")
		(*b*) Provide in the abstract an informative and balanced summary of what was done and what was found	Abstract – includes background, methods, main results, and conclusions.
**Introduction**			
Background/rationale	2	Explain the scientific background and rationale for the investigation being reported	Introduction, paragraphs 1–3.
Objectives	3	State specific objectives, including any prespecified hypotheses	Introduction, last paragraph – objective stated clearly.
**Methods**			
Study design	4	Present key elements of study design early in the paper	Materials and Methods, Section 2.1 (Study design and sample size calculation).
Setting	5	Describe the setting, locations, and relevant dates, including periods of recruitment, exposure, follow-up, and data collection	Materials and Methods, Section 2.1 – Kragujevac, Serbia; study period Sept 2024–March 2025.
Participants	6	(*a*) Give the eligibility criteria, and the sources and methods of selection of participants	Materials and Methods, Section 2.2 (Inclusion and Exclusion Criteria).
Variables	7	Clearly define all outcomes, exposures, predictors, potential confounders, and effect modifiers. Give diagnostic criteria, if applicable	Methods, Sections 2.3–2.4 – variables and reliability testing described.
Data sources/ measurement	8*	For each variable of interest, give sources of data and details of methods of assessment (measurement). Describe comparability of assessment methods if there is more than one group	Methods, Sections 2.3 (Data collection and image processing) and Tables 1–3.
Bias	9	Describe any efforts to address potential sources of bias	Section 2.4 (Measurement Reliability) – ICC used to assess intra-examiner reliability.
Study size	10	Explain how the study size was arrived at	Section 2.1 – sample size calculated using G*Power software (n=82).
Quantitative variables	11	Explain how quantitative variables were handled in the analyses. If applicable, describe which groupings were chosen and why	Section 2.5 (Statistical Analysis) – mean ± SD, tests used, treatment of variables described.
Statistical methods	12	(*a*) Describe all statistical methods, including those used to control for confounding	Section 2.5 – statistical tests (t-test, ANOVA, regression, Pearson correlation).
		(*b*) Describe any methods used to examine subgroups and interactions	Results 3.4 and Tables 8–9 – subgroup comparisons by

			gender and tooth shape.
		(*c*) Explain how missing data were addressed	Not applicable – all participants had complete datasets.
		(*d*) If applicable, describe analytical methods taking account of sampling strategy	Not applicable.
		(*e*) Describe any sensitivity analyses	Not performed – not applicable.
**Results**			
Participants	13*	(a) Report numbers of individuals at each stage of study—eg numbers potentially eligible, examined for eligibility, confirmed eligible, included in the study, completing follow-up, and analysed	Results, first paragraph – total n=82 (62 women, 20 men).
		(b) Give reasons for non-participation at each stage	Not applicable
		(c) Consider use of a flow diagram	Not applicable
Descriptive data	14*	(a) Give characteristics of study participants (eg demographic, clinical, social) and information on exposures and potential confounders	Results, Tables 4–5 (facial and dental characteristics by gender).
		(b) Indicate number of participants with missing data for each variable of interest	Not applicable – all participants had complete datasets (no missing data reported).
Outcome data	15*	Report numbers of outcome events or summary measures	Results, Tables 4–10 – mean values, SDs, correlations, regression coefficients
Main results	16	(*a*) Give unadjusted estimates and, if applicable, confounder-adjusted estimates and their precision (eg, 95% confidence interval). Make clear which confounders were adjusted for and why they were included	Results, Tables 4–7 – includes p-values and 95% CI.
		(*b*) Report category boundaries when continuous variables were categorized	Not applicable – all quantitative variables were analyzed as continuous measures (no categorization was performed).
		(*c*) If relevant, consider translating estimates of relative risk into absolute risk for a meaningful time period	Not applicable – study does not involve risk estimation or follow-up; correlational and regression analyses only.
Other analyses	17	Report other analyses done—eg analyses of subgroups and interactions, and sensitivity analyses	Results, Section 3.3–3.4 – subgroup analysis by tooth shape and gender.
**Discussion**			
Key results	18	Summarise key results with reference to study objectives	Discussion, first paragraph.
Limitations	19	Discuss limitations of the study, taking into account sources of potential bias or imprecision. Discuss both direction and magnitude of any potential bias	Discussion, 'Study limitations' subsection.
Interpretation	20	Give a cautious overall interpretation of results considering objectives, limitations, multiplicity of	Discussion, final paragraphs.

		analyses, results from similar studies, and other relevant evidence	
Generalisability	21	Discuss the generalisability (external validity) of the study results	Conclusions – limitations and applicability discussed.
**Other information**			
Funding	22	Give the source of funding and the role of the funders for the present study and, if applicable, for the original study on which the present article is based	Funding statement – Section 'Funding' at the end of the paper.

*Give information separately for exposed and unexposed groups.

Note: An Explanation and Elaboration article discusses each checklist item and gives methodological background and published examples of transparent reporting. The STROBE checklist is best used in conjunction with this article (freely available on the Web sites of PLoS Medicine at http://www.plosmedicine.org/, Annals of Internal Medicine at http://www.annals.org/, and Epidemiology at http://www.epidem.com/). Information on the STROBE Initiative is available at www.strobe-statement.org.
